# Supplementary material for: Causal interplay between lactose intolerance and gut microbiota: a combined bidirectional Mendelian randomization and in vivo validation study
Source: Front Nutr. 2026 Jun 1;13:1803337. doi: 10.3389/fnut.2026.1803337 (PMC13265576; doi:10.3389/fnut.2026.1803337)
Supplement: Supplementary file 1 [file Data_Sheet_1.zip › supplementary materials/Forward/forest plot/ebi-a-GCST90027791.finngen_R12_E4_LACTONAS.pdf]

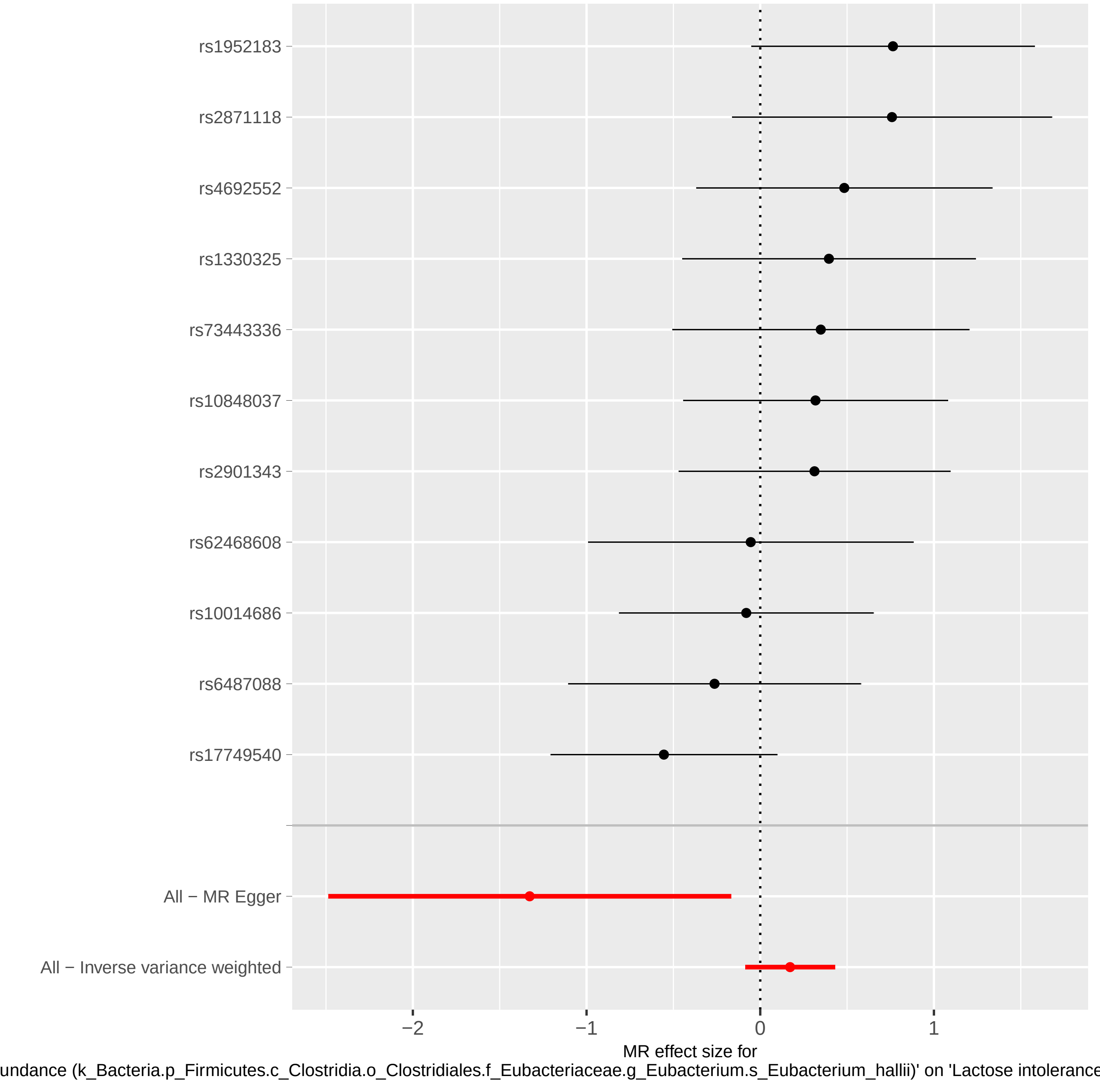
rs1952183
rs2871118
rs4692552
rs1330325
rs73443336
rs10848037
rs2901343
rs62468608
rs10014686
rs6487088
rs17749540
All − MR Egger
All − Inverse variance weighted
−2
−1
0
1
MR effect size for
undance (k_Bacteria.p_Firmicutes.c_Clostridia.o_Clostridiales.f_Eubacteriaceae.g_Eubacterium.s_Eubacterium_hallii)' on 'Lactose intolerance
